# Protein–RNA interactions: structural characteristics and hotspot amino acids

**DOI:** 10.1261/rna.066464.118

**Published:** 2018-11

**Authors:** Dennis M. Krüger, Saskia Neubacher, Tom N. Grossmann

**Affiliations:** 1 Chemical Genomics Centre of the Max Planck Society, 44227 Dortmund, Germany; 2 Department of Chemistry and Pharmaceutical Sciences, VU University Amsterdam, 1081 HV Amsterdam, The Netherlands

**Keywords:** alanine scanning, protein–RNA complex, RNA-binding protein, ribonucleoprotein, secondary structure

## Abstract

Structural information about protein–RNA complexes supports the understanding of crucial recognition processes in the cell, and it can allow the development of high affinity ligands to interfere with these processes. In this respect, the identification of amino acid hotspots is particularly important. In contrast to protein–protein interactions, in silico approaches for protein–RNA interactions lag behind in their development. Herein, we report an analysis of available protein–RNA structures. We assembled a data set of 322 crystal and NMR structures and analyzed them regarding interface properties. In addition, we describe a computational alanine-scanning approach which provides interaction scores for interface amino acids, allowing the identification of potential hotspots in protein–RNA interfaces. We have made the computational approach available as an online tool, which allows interaction scores to be calculated for any structure of a protein–RNA complex by uploading atomic coordinates to the PRI HotScore web server (https://pri-hotscore.labs.vu.nl).

## INTRODUCTION

The transcriptome encompasses a large variety of different functional RNA classes. As a common feature, most of the RNA functions rely on interactions with proteins. In addition, RNA biosynthesis and regulation is governed via numerous RNA-binding proteins (RBP). As a result, RNA is associated with proteins through most of its life cycle. Initially described RBPs often contained conserved RNA-binding domains (Jones et al. 2001; Gerstberger et al. 2014). With recent advancements in large-scale, system-wide mapping approaches, the number of known RBPs has increased to more than 4000 (Castello et al. 2016; He et al. 2016) and so has the number of RNA-binding domains that do not share sequence homology with the traditional canon of domains. Due to high-throughput X-ray crystallography and more powerful NMR instrumentation, structural information about PRIs has also increased with currently more than 2000 protein–RNA structures being available in the Protein Data Bank (PDB) (Berman et al. 2000).

Structural information about biomacromolecular complexes can foster the understanding of recognition processes and the development of high-affinity ligands (Arkin et al. 2014; Pelay-Gimeno et al. 2015). For protein–protein interactions (PPI), it was found that some amino acids contribute more to binding than others. These so-called hotspots are defined as amino acid positions where variation to alanine leads to an increase in the binding free energy (ΔG) of at least 2.0 kcal·mol^─1^ (Clackson and Wells 1995; Moreira et al. 2007) and they often overlap with conserved residues (Keskin et al. 2005). The experimental identification of hotspots via sequential alanine variations is very labor intensive which stimulated the development of computational alanine scanning approaches for PPIs (Morrow and Zhang 2012). In particular, the design of peptide-derived PPI inhibitors has benefited from this knowledge resulting in the development of numerous bioactive peptidomimetic PPI inhibitors (Pelay-Gimeno et al. 2015).

With a few exceptions RNA proves to be a very challenging target for therapeutic intervention when using small molecular scaffolds (Thomas and Hergenrother 2008; Connelly et al. 2016). In principle, available structures of protein–RNA complexes could provide valuable starting points for the design of peptidomimetic RNA inhibitors. However, compared to PPIs, protein–RNA complexes have been studied less intensively, resulting in the lack of extensive experimental alanine scanning data (Jones et al. 2001; Yi et al. 2017). Also, there are only a few computational approaches for the characterization of protein–RNA complexes (Kumar et al. 2008; Barik et al. 2015; Paz et al. 2016). Importantly, available hotspot prediction for PRIs either relies on information about the evolutionary conservation of amino acids or it is relatively time consuming (Walia et al. 2014; Barik et al. 2016; Pan et al. 2018).

Herein, we report an analysis of structures of protein–RNA complexes available via the PDB. Based on these complexes a data set of 322 crystal and NMR structures was assembled which served as basis for our analysis. After the investigation of structural features of these complexes, we describe a computational alanine-scanning approach which provides an “interaction score” for interface amino acids allowing the identification of potential hotspots in the RNA-binding region. We made this approach available via the PRI HotScore web server (https://pri-hotscore.labs.vu.nl), allowing the calculation of interaction scores for any protein–RNA complex for which atomic coordinates are available.

## RESULTS

### Data set assembly and characteristics

Initially, we assembled a data set of protein–RNA structures suitable for the investigation of PRIs. First, the PDB was searched for crystal structures with a resolution of 3 Å and below, and NMR structures with complexes in which the protein component contains greater or equal to 10 amino acids (Supplemental Fig. S1). In the resulting 1115 structures, we checked proteins in complexes with the same RNA sequence for redundancy, which reduced the number of PDB entries to 544. Among protein–RNA complexes, ribosomal PRIs represent a very particular case, as large RNA structures are permanently associated with embedded, relatively small protein chains. As a consequence, ribosomal RNA exhibits unique properties such as high structural integrity and catalytic activity. For these reasons, we decided to remove complete ribosomes from the data set. Finally, complexes involving RNA sequences with less than 10 nucleotides (nt) and more than 50% modified nucleobases were excluded resulting in the final data set of 49 NMR and 273 crystal structures (Supplemental Table S1).

**FIGURE 1.**
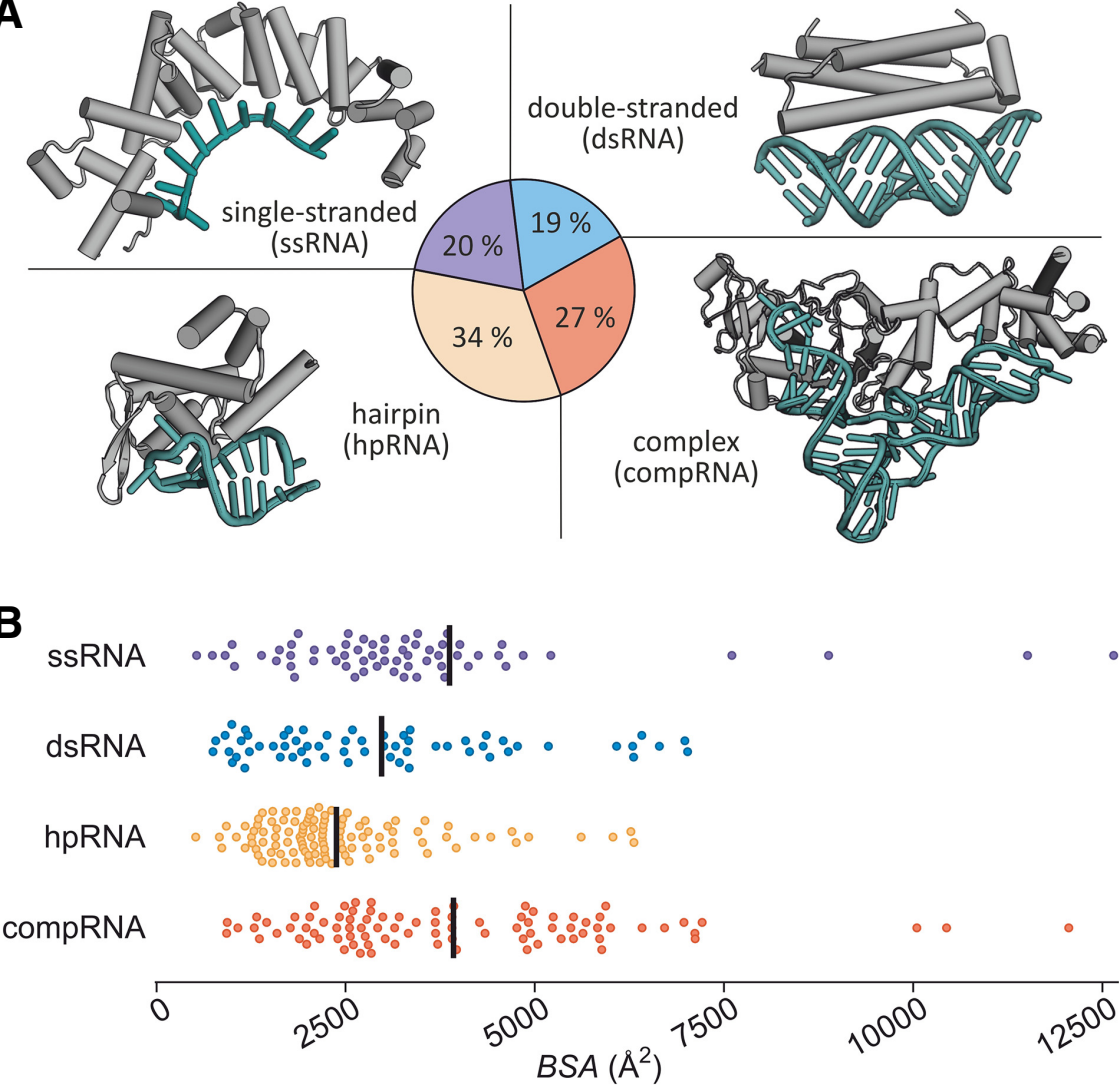
Characteristics of RNA binding motifs. (*A*) Proportion of RNA motifs in PRIs showing an example for each from the data set: ssRNA (PDB ID 3k49), dsRNA (PDB ID 2az0), hpRNA (PDB ID 4qi2), compRNA (PDB 3akz). For a complete list of PDB IDs in the data set and corresponding RNA motif classification, see Supplemental Table S1. (*B*) Scatter dot plot of BSA distribution including average values (black line). For ssRNA the two data points >13000 Å^2^ are not depicted (for all values see Supplemental Table S1).

For this data set, we first determined the buried surface area (*BSA*) providing an average of 3211 Å^2^ with most of the complexes (80%) ranging between 1400 and 6000 Å^2^. In contrast, interfaces in PPIs have been reported to range between 600 and 4700 Å^2^ (Lo Conte et al. 1999; Luo et al. 2015). Next, the 322 complexes in our data set were grouped based on involved RNA structural motifs using the following categories: single-stranded RNA (ssRNA, 20%), double-stranded RNA (dsRNA, 19%), hairpin RNA (hpRNA, 34%), and RNA with complex folds (compRNA, 27%) (Fig. 1A; Supplemental Table S1). A comparison of these groups (Fig. 1B) reveals that hpRNAs (yellow) exhibit the smallest average PRI interfaces (mean *BSA* = 2377 Å^2^) and a relatively narrow *BSA* distribution (Fig. 1B). Broader BSA distributions are observed for the other three classes. CompRNA (red) and ssRNA (violet) provide the largest average interfaces (mean *BSA* = 3931 Å^2^ and 3829 Å^2^, respectively) followed by dsRNA (blue, mean *BSA* = 2976 Å^2^).

**FIGURE 2.**
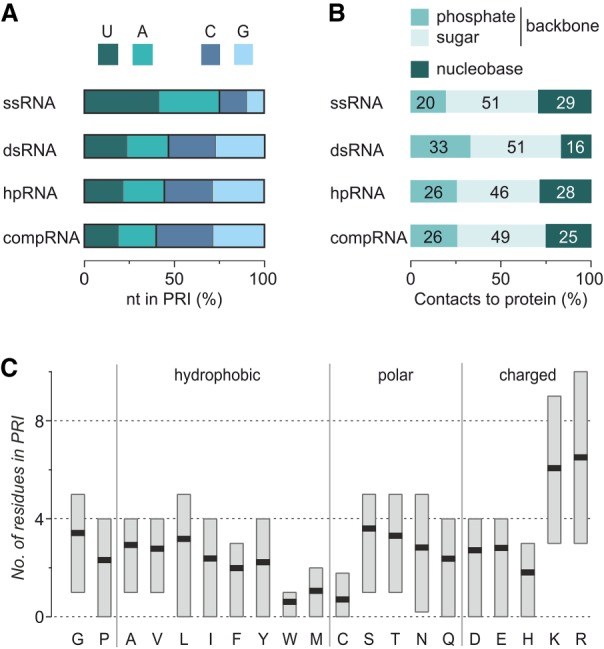
PRI interface properties. (*A*) Average RNA nucleotide frequency in the PRI interface. (*B*) Average contribution of RNA backbone (phosphate and sugar) as well as nucleobase atoms to PRI contacts (nt: nucleotide). (*C*) Average amino acid frequency (black lines) in PRI interfaces. Boxes represent the 60% core distribution (ranging from 20th to 80th percentile) of the number of each amino acid per PRI interface.

Next, we analyzed the characteristics of the protein-bound RNA sequences in detail. Averaging over all complexes in the data set, there is no preference for certain nucleotides (nt) in the PRI (mean frequency of each nt = 4.4–5.0, Supplemental Fig. S2). For dsRNA and hpRNA, nucleotides are relatively evenly distributed (Fig. 2A) resulting in similar A/U and G/C ratios which owes to the importance of Watson–Crick base paring in these structural motifs. Complexes with ssRNA on the other hand show less balanced A/U (1:1.3) as well as G/C (1.6:1) ratios, and a relatively high content of A and U (72%). The latter is a result of the preferred use of poly(A) or poly(U) sequences for cocrystallization with nonsequence specific RNA-binding domains. The lowest AU content (40%) in PRI interfaces is found for compRNA, however, exhibiting balanced A/U and G/C ratios (Fig. 2A). When analyzing the contacts between RNA and protein, we observe a preference for contacts via RNA backbone atoms (mean contribution of backbone atoms = 72%–87% comparing all four RNA motifs) opposed to RNA base atoms (Fig. 2B). When comparing the different RNA motifs, we observe the highest backbone involvement for dsRNA (mean contribution: 87%) resulting from the shielding of bases in the RNA duplex. Notably in some cases, contacts via RNA bases can be considerably higher in particular when single-stranded RNA stretches are recognized in a sequence specific manner. One example is the Puf3 protein from yeast that binds a conserved sequence in 3′-UTRs (Fig. 1A ssRNA, PDB ID 3k49) (Zhu et al. 2009) and facilitates 39% of the contacts to RNA via the nucleobases.

After analyzing the interacting RNA, we turned toward the protein. Notably, the type of protein secondary structure involved in RNA recognition is relatively independent of the bound RNA structural motif and shows some preference for helixes (Supplemental Fig. S3). On average, 56 amino acids are involved per PRI, with 60% of all structures revealing between 26 and 80 interface amino acids. Analysis of the amino acid composition in the PRI interface shows a clear preference for lysine (K) and arginine (R) with an average occurrence of 6.1 and 6.5 residues per PRI, respectively (Fig. 2C). The high frequency of these positively charged amino acids is in line with the fact that on average 26% of the contacts in protein–RNA interactions occurs via the negatively charged RNA phosphates. Most of the other amino acids occur on average between 1.8- and 3.3-times per interface with the exception of tryptophan (W, 0.6-times), methionine (M, 1.1-times) and cysteine (C, 0.7-times) which show lower frequencies. These lower frequencies are in line with the overall low occurrence of these three amino acids in proteins (Brooks et al. 2002; Talavera et al. 2011).

Overall amino acid frequencies are dominated by interactions between the protein and the RNA backbone (Fig. 2B) explaining the high occurrence of arginine (R) and lysine (K). To evaluate the amino acid involvement in nucleobase binding, we next focused exclusively on protein–nucleobase contacts (Supplemental Fig. S4). Analyzing the four bases separately, we found arginine (R) to be heavily involved in interactions with all bases, contributing with 16%–20% of all contacts while lysine (K) only provides 3%–9%. Other than arginine (R), only phenylalanine (F) and tyrosin (Y) contribute to ≥10% of the protein–nucleobase contacts: U shows a particularly high involvement (F: 16%, Y: 17%) followed by A (F: 10%, Y: 9%) and G (F: 8%, Y: 11%). For the remaining amino acids, we do not observe substantial deviations from overall interface frequency.

### Virtual alanine scanning for hotspot prediction

To evaluate the contribution of interface amino acids to binding, we aimed for the development of an in silico alanine scanning workflow enabling the analysis of protein–RNA structures. To ensure accurate predictions as well as short calculation times, we considered the use of knowledge-based potentials which sum over individual pairwise interaction terms of interface residues and have proven useful for in silico alanine scanning in protein–protein complexes (Krüger and Gohlke 2010). We selected the Decoys As the Reference State potential (DARS-RNP) (Chuang et al. 2008), which considers interactions in a distance- and orientation-dependent manner and has been used for scoring of different decoys and conformations of protein–RNA complexes (Tuszynska and Bujnicki 2011). DARS-RNP shows very good performance in identifying native-like structures, and it is able to score RNA sequences with post-transcriptionally modified nucleobases (Tuszynska and Bujnicki 2011). The original DARS-RNP training data comprised 44 protein–RNA complexes of which 22 are also part of our 322 complexes containing a data set (for PDB IDs see Materials and Methods section).

We determined DARS scores for the 322 starting complexes and for 13,780 complexes resulting from the variation of each interface amino acid in these complexes to alanine. In this analysis, a distance of 7 Å was used as threshold, which was shown to be the optimal cut-off for analyzing protein–nucleic acid interactions with such potentials (Donald et al. 2007). Glycine as well as proline (and of course alanine itself) were not varied (Moreira et al. 2007; Morrow and Zhang 2012). Next, the difference between the natural logarithm of DARS scores of the starting and an alanine varied complex were calculated to provide Δln(*DARS*) values, which are a measure for the contribution of each interface amino acid for RNA-binding: Large values indicate a great loss in binding affinity upon alanine substitution. Notably, for 19.6% of the interface amino acids we obtain negative Δln(*DARS*) scores indicating considerable involvement of these residues in intramolecular contacts (Krüger and Gohlke 2010; Tuszynska and Bujnicki 2011). For subsequent calculations these residues were neglected. To ensure comparability between different complexes, the remaining 11107 Δln(*DARS*) scores were normalized per interface to provide the final interaction score (*IS*). As a consequence of the normalization, the average of all *IS* values per interface equals 1.

**FIGURE 3.**
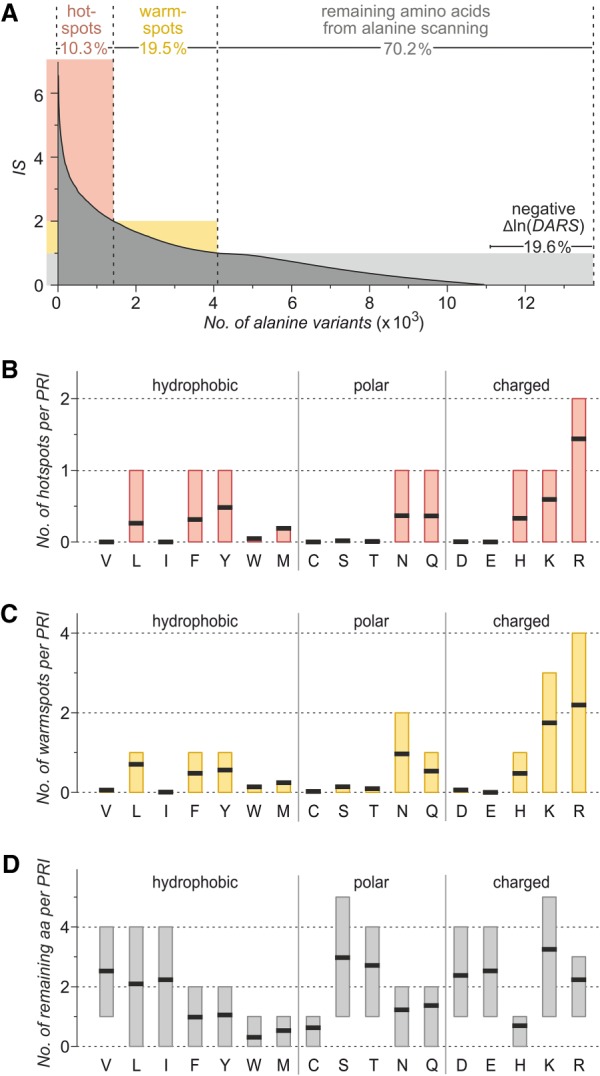
Analysis of in silico alanine scanning. (*A*) Distribution of interaction scores (IS) for interface amino acids in the 322 protein–RNA complexes. Glycine, proline, and alanine were not considered during alanine scanning. A total of 2699 alanine variants (19.6%) provide negative Δln(*DARS*) scores and were not included in the calculation of *IS* values. The *IS* range of hotspots (red, *IS* > 2) and warmspots (orange, 2 ≥ *IS* > 1) is indicated. (*B*–*D*) Average amino acid frequency (black lines) in PRI interfaces considering hotspots (*B*, *IS* > 2, red), warmspots (*C*, 2 ≥ *IS* > 1, orange), and the remaining interface residues (*D*, gray) considered during in silico alanine scanning. Boxes represent the 60% core distribution (ranging from 20th to 80th percentile) of the number of the corresponding amino acid per PRI interface.

Overall, we observed a distribution of *IS* values between 0 and 6.62 (Fig. 3A) with 29.8% of all residues considered during alanine scanning providing scores above average (*IS* > 1). Only 10.3% of considered residues obtain scores that are twofold over average (*IS* > 2). Since this occurrence is similar to the frequency of hotspots in PPIs (9.5%) (Moreira et al. 2007), we defined residues with *IS* > 2 as hotspots. Notably, amino acid frequencies of these hotspots per PRI (Fig. 3B) differ considerably from the general interface distribution of amino acids (Fig. 2C). We observe a preference for arginine (R) over lysine (K) and an enrichment of leucine (L), phenylalanine (F), tyrosine (Y), asparagine (N), glutamine (Q), and histidine (H). The strong preference of arginine (R) is not surprising given its ability to form multiple hydrogen bonds and salt-bridges with electron-deficient groups such as the RNA backbone phosphates (Bogan and Thorn 1998). Interestingly, the amino acid distribution of residues with an interaction score between 1 and 2 (2 ≥ *IS* > 1) is similar to the one found for PRI hotspots (*IS* > 2) (Fig. 3C). Based on this observation, we termed these residues “warmspots” as a considerable contribution to binding can be expected from these amino acids. It is noteworthy that out of the 322 complexes in the data set, 292 harbor at least one residue with *IS* > 2 (hotspot) but all complexes contain residues with *IS* > 1 (hotspot and/or warmspot). Compared to hot- and warmspots, the remaining residues (*IS* ≤ 1, 70.2%) show very different amino acid frequencies (Fig. 3D), which are similar to the general interface distribution (Fig. 2C) but showing the expected reduction in hot- and warmspot amino acids.

### Experimentally validated hotspots

Experimental data about the contribution of interface amino acids to the stability of protein–RNA complexes is limited since studies often focus on a variation of the RNA rather than the protein sequence. Among the complexes in our data set, we identified two examples with considerable data on alanine substitution of interface residues. One is the complex of nucleolar essential protein 1 (Nep1, PDB ID 3oij) with its target ribosomal RNA (Thomas et al. 2011), and the other a complex between a bacterial RNA-binding carbon storage regulator (CsrA, PDB ID 2jpp) and its messenger RNA recognition fragment (Schubert et al. 2007). Importantly, none of these complexes is part of the original DARS-RNP training data set.

**FIGURE 4.**
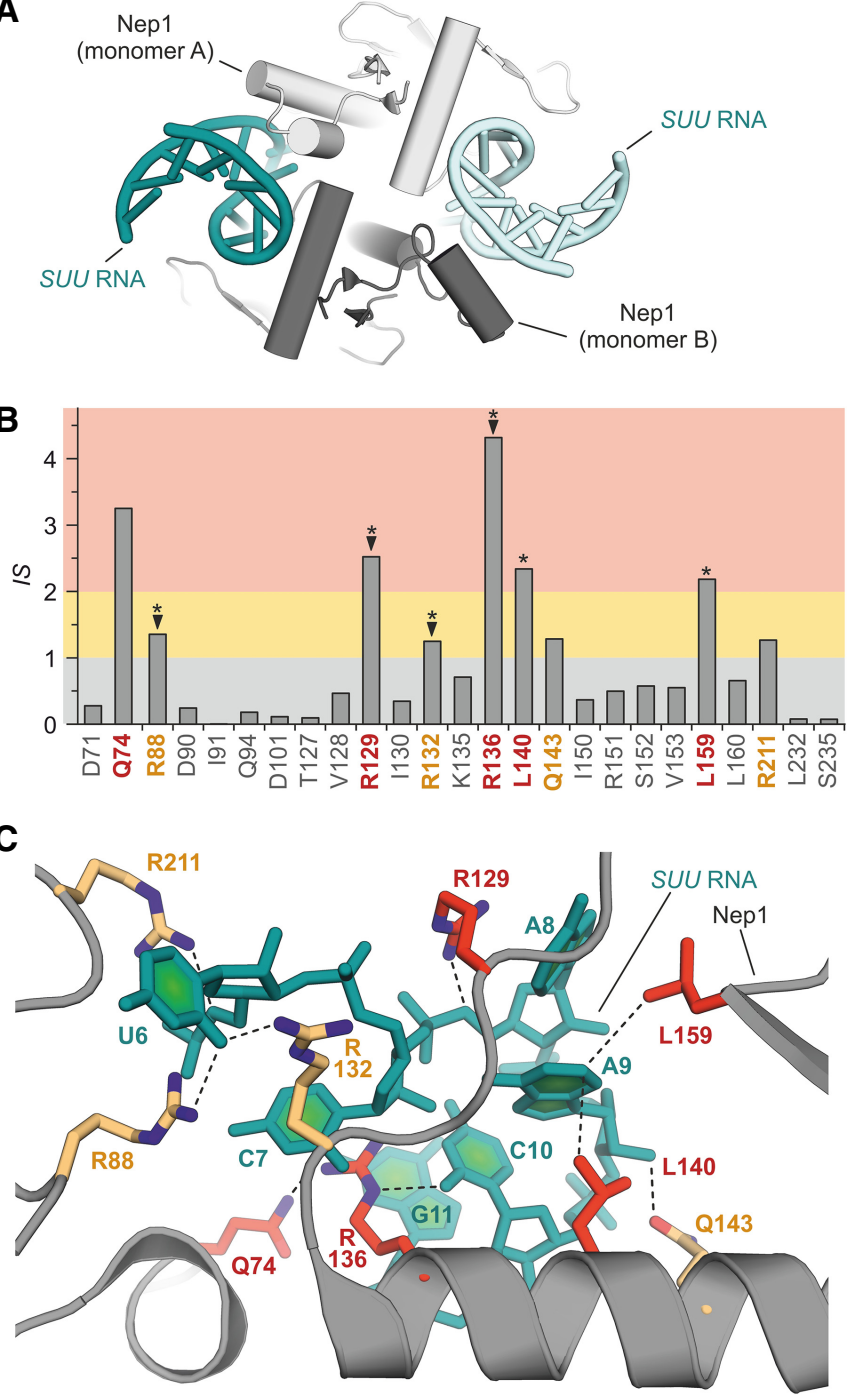
Analysis of crystal structure of Nep1 with *SUU* rRNA (Thomas et al. 2011). (*A*) Crystal structure in cartoon representation of the Nep1/*SUU* RNA complex (PDB ID 3oij). (*B*) Interaction scores (*IS*) for Nep1 amino acids in contact with *SUU* RNA (based on PDB ID 3oij). Hotspots (red) and warmspots (orange) are highlighted. ([▾] Hotspots confirmed via yeast three-hybrid system [Taylor et al. 2008], [*] conserved residues [Thomas et al. 2011]). (*C*) Detailed view of the PRI of Nep1/*SUU* RNA with hotspots (red) and warmspots (orange) explicitly. For each highlighted amino acid, closest protein–RNA contacts (between heavy atoms) are shown as dashed line.

The cocrystal structure of Nep1 from *Saccharomyces cerevisiae* and a fragment of the small subunit rRNA reveals a C2-symmetric Nep1 homodimer bound to two identical 14-mer RNA hairpins (PDB ID 3oij, Fig. 4A; Thomas et al. 2011). The interface between one RNA hairpin and Nep1 exhibits a *BSA* of 2162 Å^2^. Our computational workflow identifies 35 amino acids for alanine scanning. Ten of these residues obtain negative Δln(*DARS*) values and the remaining 25 amino acids provide *IS* values between 0 and 4.32 (Fig. 4B). Among those, there are five predicted hotspot (red: Q47, R129, R136, L140, L159) and four warmspot residues (orange: R88, R132, Q143, R211). The crystal structure shows that the two leucine side chains (L140 and L159) pack against the hydrophobic π-interface of nucleobases (A8 and C10, respectively) while the glutamine and arginine residues interact with backbone phosphates and/or the Watson–Crick interface of nucleobases (Fig. 4C). Prior to crystallization, four arginine-to-alanine *Nep1* mutants (R88A, R129A, R132A, R136A) were tested in a yeast three-hybrid system showing for all variants a complete loss in their ability to bind the target RNA (▾ in Fig. 4B; Taylor et al. 2008). Importantly, two of these validated hotspots are predicted hotspots (R129, R136) and two are predicted warmspots (R88, R132). Since additional alanine scanning data is not available, we compared our interaction scores with evolutionary conserved residues which, in PPIs, often overlap with hotspots (Keskin et al. 2005). Strikingly, among the interface residues all conserved amino acids ([*] in Fig. 4B) indeed overlap with our predicted hotspots (R129, R136, L140, L159) and warmspots (R88, R132). In another study, interface glutamate D90 was replaced by a glycine due to the implications of this mutation in the Bowen–Conradi syndrome (Meyer et al. 2011). Importantly, the D90G variant exhibits increased RNA affinity, which is in line with the low score for D90 (*IS* = 0.24) suggesting only small or no contributions to RNA binding by the glutamate side chain.

**FIGURE 5.**
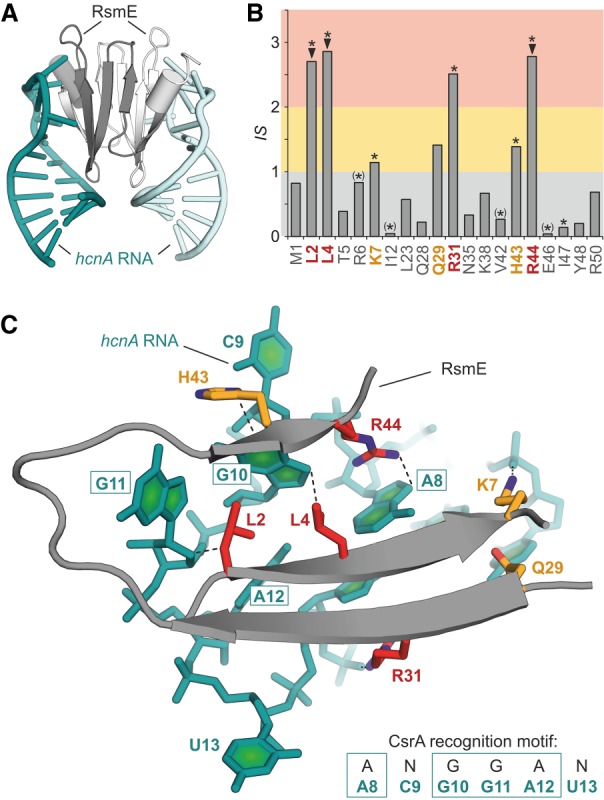
Analysis of PRI between RsmE and *hcnA* RNA (Schubert et al. 2007). (*A*) NMR structure in cartoon representation of the RsmE/*hcnA* RNA complex (PDB ID 2jpp). (*B*) Hotspot scores for RsmE residues in contact with *hcnA* RNA (based on PDB ID 2jpp). Hotspots (red) and warmspots (orange) are highlighted ([▾] Hotspots confirmed via ITC [Mercante et al. 2006], [*] in vivo regulatory defect [≥40%, associated with lack of RNA binding] upon alanine variation [^(*)^] residues with intradomain interactions) (Mercante et al. 2006). (*C*) Detailed view of the PRI of RsmE/*hcnA* RNA with hotspots (red) and warmspots (orange) shown explicitly. For each highlighted amino acid, closest protein–RNA contacts (between heavy atoms) are shown as dashed line.

A second structure in our data set for which we found affinity data of alanine variants is the complex between a CsrA protein and its mRNA recognition element. CsrA proteins can be found in numerous bacteria and contain a conserved RNA-binding domain (Mercante et al. 2006). Here, we analyze the NMR solution structure of an CsrA homolog, RsmE, in complex with its RNA target site originating from the *hcnA* RNA transcript (PDB ID 2jpp, Fig. 5A; Schubert et al. 2007). In this structure, RsmE forms a homodimer which binds to two RNA recognition elements providing a *BSA* of 2030 Å^2^ for each of them. One PRI contains 29 interface residues which were considered for alanine scanning with nine of them providing negative Δln(*DARS*) values. The interaction score for the remaining 20 residues ranges between 0.04 and 2.86 (Fig. 5B) resulting in four predicted hotspots (red: L2, L4, R31, R44) and three warmspots (orange: K7, Q29, H43). The NMR structure reveals that hotspot residues L2, L4, and R44 directly contact the conserved nucleobases in the RNA target sequence (A N G G A N) while the other hot- and warmspots have contacts with the RNA backbone (Fig. 5C).

In a study that predates the NMR structure, an alanine-scan of the RNA-binding domain of a very closely related CsrA ortholog from *Escherichia coli* (for sequence alignment see Supplemental Fig. S5) was performed using a phenotypic readout (Mercante et al. 2006). Focusing on a regulatory defect associated with an impaired CsrA–RNA interaction, a number of inactive alanine-variants were identified. Among those, the amino acids that are involved in intradomain interactions ([*] in Fig. 5B) exhibit low *IS* values. Strikingly, when considering the alanine-variants that are not involved in such intramolecular interactions (* in Fig. 5B), we observe an excellent overlay with hotspot and warmspot residues. One warmspot (Q29) does not show up in the phenotypic screen while another variant is inactive (I47A) in the assay but was not predicted to be important for binding. In the same study, a subset of alanine-variants was investigated regarding their RNA affinity using isothermal titration calorimetry (Mercante et al. 2006). These measurements verified a substantial decrease in RNA affinity for three alanine variants (ΔG ≥ 2.0 kcal · mol^─1^): L2A (73-fold), L4A (62-fold), R44A (150-fold). Importantly, all of these residues are predicted hotspots.

For further validation, we made use of the database of Alanine Mutagenic Effects for Protein–Nucleic Acid Interactions (dbAMEPNI), which contains experimental ΔΔG-values resulting from alanine subsitution for 50 protein–RNA complexes (Liu et al. 2018). In the PRI HotScore algorithm, *IS* values are normalization per interface, which hampers the comparison of residues of different unrelated complexes. For this reason, we searched for complexes in dbAMEPNI that provide at least five pairs of experimental *ΔΔG* and *IS* values. This search yielded seven complexes with five to maximally 20 data pairs (Table 1; Supplemental Table 2). Most notably, for five of these complexes, we obtain a good Pearson correlation coefficient (*r* = 0.49–0.77). One of the complexes (PDB ID 1jbs, *r* = 0.77) is part of the original DARS training set, which may explain its good correlation. However, here it is important to note that the generation of the DARS-RNP did not involve information about experimental alanine scanning but only considered structural data (Chuang et al. 2008).

**TABLE 1.**
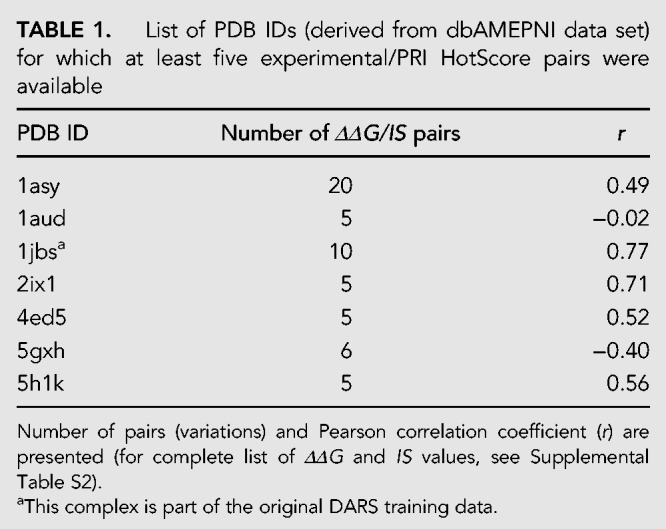
List of PDB IDs (derived from dbAMEPNI data set) for which at least five experimental/PRI HotScore pairs were available

## DISCUSSION

Based on the structures of protein–RNA complexes available through the PDB, we assembled a data set of 322 complexes which provides a relatively even distribution between single-stranded RNA (20%), duplexes (19%), hairpins (34%), and more complex folds (27%). Overall, the size of the interaction area (*BSA*) in PRIs appears to be larger than in PPIs which may account for the generally higher flexibility of RNA-structures (Hermann 2002) when compared to folded protein domains. This can cause entropic penalties upon binding and rigidification and thereby affect binding affinities (Pelay-Gimeno et al. 2015). Also, protein–RNA interfaces bear significantly more positively charged amino acids (arginine and lysine) than PPIs, which can be explained by the considerable involvement of the RNA backbone phosphates in PRIs.

To enable the prediction of amino acid hotspots in known protein–RNA structures, a computational workflow was established using the knowledge-based DARS potential. Our approach was implemented into the PRI HotScore web server, which is publicly available (https://pri-hotscore.labs.vu.nl) and allows the scoring of individual amino acid side chains regarding their contribution to a PRI. Importantly, PRI HotScore is independent of information regarding the conservation of amino acids, and it is computationally not demanding. However, the latter comes at the cost of simplifications such as the exclusion of water molecules and the consideration of rigid protein and RNA structures. In addition and similar to analogous alanine scanning approaches for PPIs, RNA targeting amino acids that are also involved in intradomain interactions receive a lower score which can lead to false-negative results. However, these simplifications result in a fast algorithm that recapitulates the experimental data for two test complexes very well.

PRI HotScore provides an interaction score (IS) for every interface amino acid which contributes to RNA binding [positive Δln(*DARS*) value] except for glycine, proline, and alanine. *IS* values are normalized per interface, resulting in a mean *IS* of 1 when averaging all considered interface residues. Notably, amino acids with an interaction score above average (*IS* > 1, 29.8% of alanine-scanned amino acids) exhibit a distinct pattern with high frequencies of arginine (R) and a relatively high occurrence of seven additional amino acids (L, F, Y, N, Q, H, K). Interestingly, this is very different from PPI hotspots which are mainly composed of tryptophan (W, 21%), arginine (R, 13%), and tyrosine (Y, 12%) (Lichtarge et al. 1996; Bogan and Thorn 1998). Another interesting observation is the preference of leucine (L) over isoleucine (I) in our predicted hotspots, in particular when considering that hotspots in PPIs show the opposite effect with isoleucine (I, 9.6%) occurring more than 10 times more frequently than leucine (L, 0.83%) (Bogan and Thorn 1998). These differences may originate from the fact that in PPIs residues often coevolve across the protein–protein interface (Goh and Cohen 2002; Mesa et al. 2003) which is not that straightforward in protein–RNA interfaces. For residues with an *IS* > 1, we decided to distinguish between hotspots (*IS* > 2) and warmspots (2 ≥ *IS* > 1) comprising 10.3% and 19.5% of all alanine-scanned amino acids, respectively. We introduced this separation since interaction scores twofold over average (*IS* > 2) can be expected to be significantly increased and due to the fact that about 10% of interface residues in PPIs are also considered hotspots (Moreira et al. 2007). In the two protein–RNA complexes of our data set that we considered in detail (Figs. 4 and 5), eight of the nine predicted hotspots were either experimentally validated or conserved, while this was true for five of the seven warmspots. In addition, an analysis of a sub-set of the dbAMEPNI data base provided a good correlation of experimentally determined *ΔΔG* and *IS* values for five of the seven complexes.

Future investigations will show if the discrimination between hotpots and warmspots is reasonable or if PRIs rely on more crucially interacting residues than PPIs. Also it will be of interest, how hotspot regions are distributed over the RNA interface considering that in PPIs often hotspots of both binding partners pack against each other (Moreira et al. 2007). Importantly, predicted structures of protein–RNA complexes provide an additional source for PRI HotScore alanine-scanning since the modeling of such complexes give access to structurally uncharacterized PRIs (Dawson and Bujnicki 2016). Taken together, PRI HotScore allows the identification of amino acids crucially involved in RNA recognition, which can support the future design of RNA targeting peptidomimetics.

## MATERIALS AND METHODS

### Data set

The PDB database (Berman et al. 2000) web filter was used to obtain PDB IDs for all X-ray and NMR structures that contain both protein and RNA (as of June 2017), whereas the protein sequence must be equal to or larger than 10 amino acids. For each PDB ID, the corresponding 3D coordinates were downloaded in PDB format as well as the sequence data in FASTA format. The sequence data was used to remove all PDB structures in which the proteins have a sequence identity of greater than 90%, for the same RNA binding partner, using T-Coffee (version 11.00.8cbe486) (Tommaso et al. 2011). Next, we used in-house Python scripts and manual inspection to remove all complexes that contain whole ribosomes or where the RNA sequence is shorter than 10 nt. Finally, all structures were removed in which the RNA harbors more than 50% of modified nucleobases since these are not considered by the subsequently applied DARS-RNP. The following 21 complexes (PDB IDs) are part of this data set and the original DARS-RNP training data: 1a34, 1c0a, 1di2, 1ec6, 1f7u, 1feu, 1ffy, 1j1u, 1jid, 1k8w, 1lng, 1mji, 1ooa, 1r3e, 1r9f, 1rc7, 1sds, 1u0b, 1urn, 1wsu, 1yvp.

### Characteristics of protein–RNA interface

Protein–RNA interface statistics were carried out using in-house Python scripts using the protein and RNA sequences as provided in the PDB file. For the identification of interacting amino acids and nucleotides, a 4 Å cut-off was applied. The calculation of solvent accessible surfaces (*BSA*) were performed using PyMOL (Version 1.7.7.1, Schrödinger, LLC). Secondary structure determination was performed with DSSP (version 2.0.4) (Kabsch and Sander 1983; Touw et al. 2015).

### In silico alanine scanning

An interface was defined as all amino acids within 7 Å distance from the RNA (Donald et al. 2007). DARS-RNP scores (Chuang et al. 2008; Tuszynska and Bujnicki 2011) were determined for the 322 starting complexes (*DARS*_*WT*_) and for the 13780 complexes resulting from the variation of each interface amino acid to alanine (*DARS*_*Ala*_) in these complexes. Alanine variations were performed with PyMOL (Version 1.7.7.1, Schrödinger, LLC). Glycine and proline as well as alanine itself were not varied (Moreira et al. 2007; Morrow and Zhang 2012). Next, the differences of the natural logarithm of DARS scores were calculated as

(1) $$\Delta \ln(DARS)=\ln(|DARS_{WT}|)-\text{ln(}|DARS_{Ala}|).$$

The Δln(*DARS*) scores provide a measure for the contribution of each interface amino acid for RNA-binding with large values indicating a great loss in binding affinity upon alanine substitution. Since negative Δln(*DARS*) scores indicate considerable involvement of the corresponding residues in intramolecular contacts (Krüger and Gohlke 2010; Tuszynska and Bujnicki 2011) these alanine variants were neglected for subsequent calculations. To ensure comparability between different complexes, the remaining 11,107 Δln(*DARS*) scores were normalized per interface. Initially, the average of Δln(*DARS*) values per interface was determined as

(2) $$\bar{{\text{Δ}}_{j}}=\sum_{i-n}\frac{\text{Δln}{\left(DARS\right)}_{i,j}}{n_{j}},$$

where *n* is the number of alanine variants [*i*, with a positive Δln(*DARS*) score] per interface (*j*). Subsequently, each Δln(*DARS*) value was divided by the average score of its interface ($\bar{\Delta_{j}}$) to provide the normalized interaction score (*IS*) as

(3) $$IS_{i,j}=\frac{\text{Δln}{\left(DARS\right)}_{i,j}}{\bar{{\text{Δ}}_{j}}}.$$

Residues with *IS* > 2 were defined as hotspots and residues with 2 ≥ *IS* > 1 as warmspots. *IS* values can be calculated for any protein–RNA complex if atomic coordinates (PDB format) are available using the PRI HotScore web server (https://pri-hotscore.labs.vu.nl).

## SUPPLEMENTAL MATERIAL

Supplemental material is available for this article.

## Supplementary Material

Supplemental Material
