# Supplementary material for: Protein–RNA interactions: structural characteristics and hotspot amino acids
Source: RNA. 2018 Nov;24(11):1457–65. doi: 10.1261/rna.066464.118 (PMC6191724; doi:10.1261/rna.066464.118)
Supplement: Supplemental Material [file supp_24_11_1457__index.html]

Protein-RNA Interactions: Structural Characteristics and Hotspot Amino Acids — Protein–RNA interactions: structural characteristics and hotspot amino acids — Supplemental Material 

# Protein–RNA interactions: structural characteristics and hotspot amino acids

## Supplemental Material

- Supplemental\_Information.pdf
