# Supplementary material for: Protein–RNA interactions: structural characteristics and hotspot amino acids
Source: RNA. 2018 Nov;24(11):1457–65. doi: 10.1261/rna.066464.118 (PMC6191724; doi:10.1261/rna.066464.118)
Supplement: Supplemental Material [file supp_066464.118_Supplemental_Information.pdf]

Supplemental Information

Protein–RNA Interactions: Structural Characteristics and Hotspot Amino Acids

Dennis M. Krüger,[1#] Saskia Neubacher,[2] and Tom N. Grossmann[1,2*]

[1] Chemical Genomics Centre of the Max Planck Society, Otto-Hahn-Str. 15, 44227 Dortmund, Germany

[2] Department of Chemistry and Pharmaceutical Sciences, VU University Amsterdam, De Boelelaan 1083, 1081 HV Amsterdam, The Netherlands

[#] Present address: Department for Epigenetics and Systems Medicine in Neurodegenerative Diseases, German Center for Neurodegenerative Diseases (DZNE), Göttingen, Germany

[*]Corresponding author: t.n.grossmann@vu.nl

1 Supplemental Figures

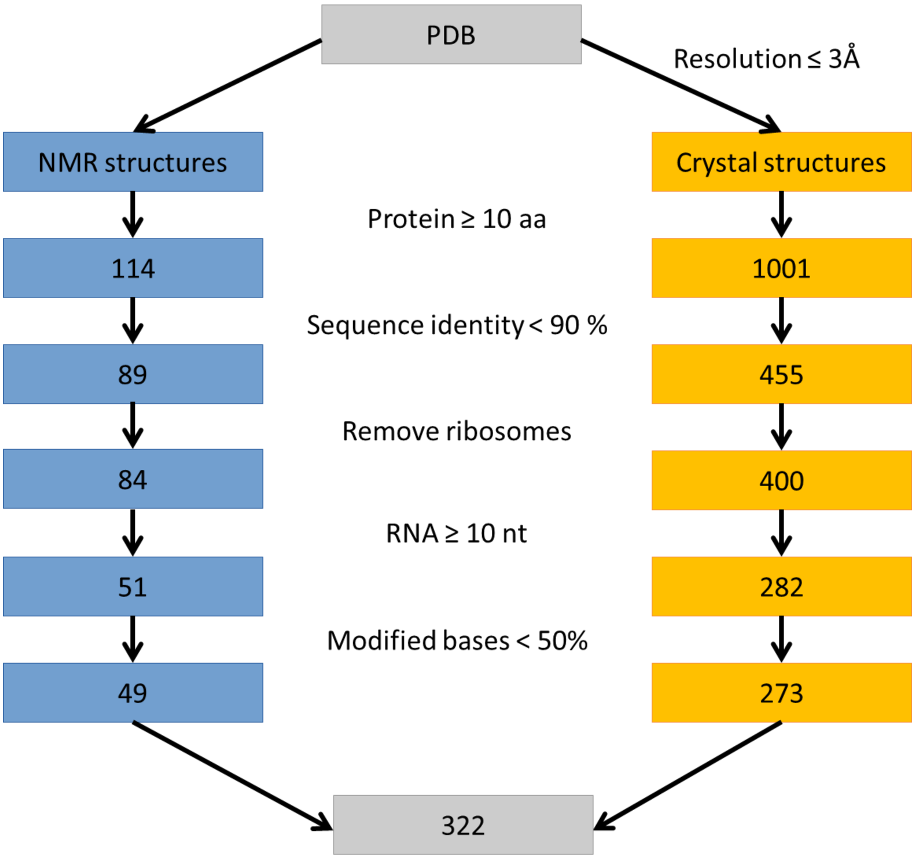

Supplemental Figure S1. Dataset preparation (August 2017).

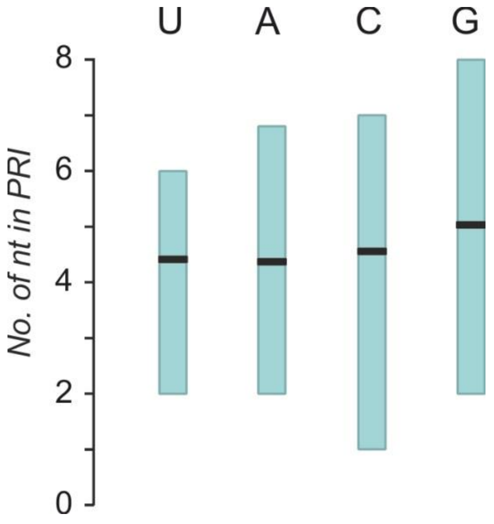

Supplemental Figure S2. RNA nucleotide (nt) frequency in PRI interfaces. Black line represents average and boxes represent the distribution of the core 60 % of occurrence in the interface (ranging from 20th to 80th percentile).

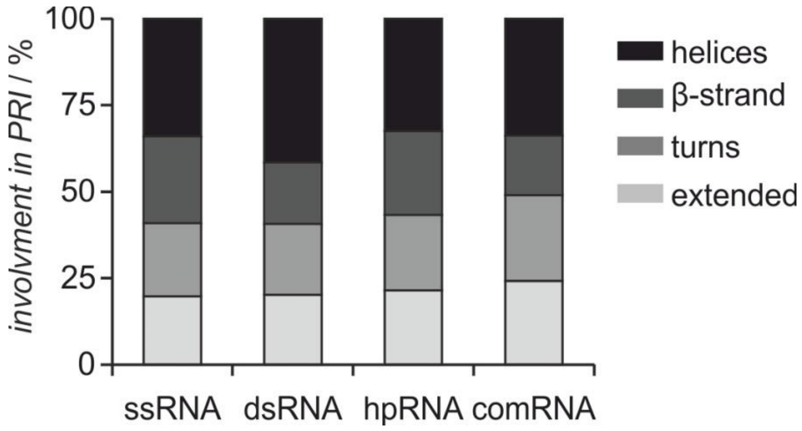

Supplemental Figure S3. Average occurrence of the different protein secondary structures (helices, β-strand, turns and extended motifs) for the four RNA structural motifs.

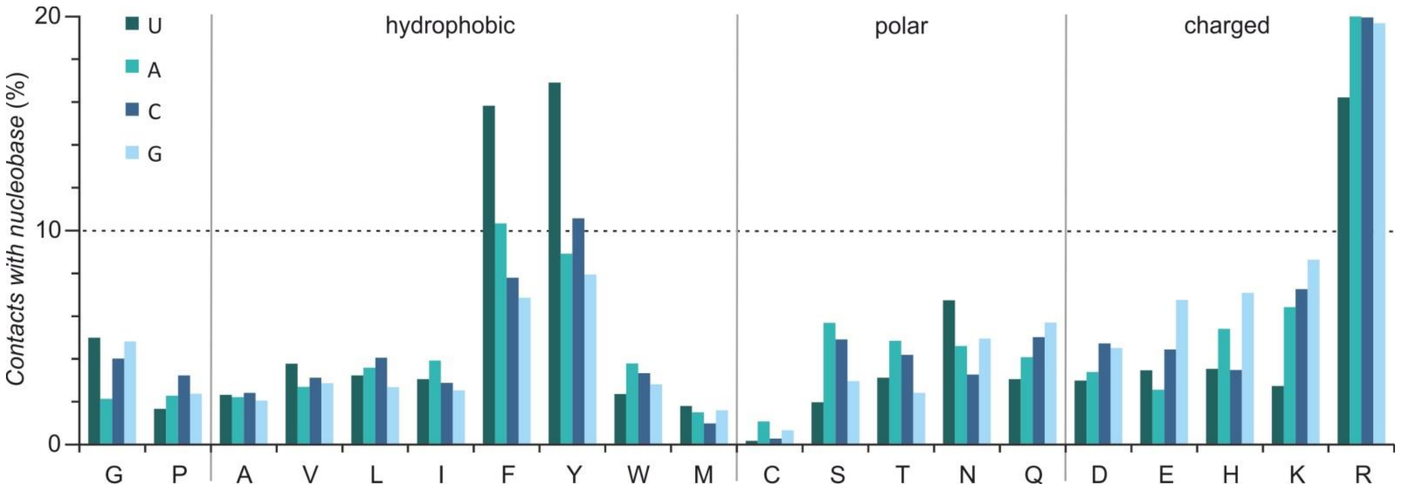

Supplemental Figure S4. Average contribution of each amino acid to nucleobase contacts is shown. Contacts were analyzed separately for each base (U, A, C, G).

```
                   1          11         21         31         41
RmsE (P. fluor.)   MLILTRKVGE SINIGDDITI TILGVSGQQV RIGINAPKDV AVHREEIYQR
CsrA (E. coli)     MLILTRRVGE TLMIGDEVTV TVLGVKGNQV RIGVNAPKEV SVHREEIYQR
                   ******.*** .. ***..*. *.*** *.** ***.****.*  *********
```

Supplemental Figure S5. Sequence alignment the RNA binding domains of RmsE form *Pseudomonas fluorescens* and CsrA from *Escherichia coli*. *In silico* alanine-scanned residues in RmsE are highlighted: hotspots (red), warmspots (yellow), residues with *IS* < 1 (grey). Positions with identical (*) and similar (.) residues are marked.

2 Supplemental Table

Table S1. List of PDB IDs included in the final dataset of 322 complexes.

ssRNA		dsRNA		hpRNA		compRNA	
**PDB ID**	**BSA / Å**	**PDB ID**	**BSA / Å**	**PDB ID**	**BSA / Å**	**PDB ID**	**BSA / Å**
2bbv	529.03	5d0a	749.23	2b6g	519.87	2rsk	929.19
4ftb	739.76	3adl	783.48	1wsu	833.08	3v7e	934.80
1sds	908.30	1f8v	908.95	4m4o	861.63	2dlc	1075.86
2bx2	997.91	3vyx	961.38	1aq3	923.08	1j1u	1290.35
2xbm	1030.55	3o3i	994.65	2f8k	1056.76	5g4u	1323.36
2py9	1389.96	2gxb	1004.82	2ese	1170.93	1u6p	1362.21
1wmq	1586.12	3fte	1056.22	1ekz	1170.99	5de5	1457.81
2adc	1626.05	1a34	1118.01	1hji	1256.01	2du3	1593.08
3boy	1771.29	4ngb	1166.89	1nyb	1266.14	2fk6	1769.37
4zlr	1777.43	2f8s	1176.31	2ann	1289.47	3cul	1829.77
4jvy	1783.97	3ks8	1206.84	4bw0	1315.32	2la5	1888.35
4u8t	1824.76	3lrr	1229.44	2pxk	1337.89	1dz5	1976.92
5e08	1872.56	4fvu	1360.66	1rlg	1344.53	1m5k	1986.48
4c9d	2077.32	3wbm	1543.57	1zse	1349.69	3moj	2027.28
1yty	2083.76	4jk0	1636.28	1e7k	1398.32	3irw	2085.41
4z0c	2306.39	1yyw	1683.22	1qfq	1398.90	1drz	2088.63
3i5x	2375.00	2mkn	1697.42	2pjp	1407.96	1hc8	2158.05
2asb	2484.88	3vyy	1748.38	1dul	1414.08	5do4	2211.21
3r2c	2500.29	4erd	1800.96	1a4t	1514.86	4ycp	2380.86
1k1g	2507.28	1dfu	1844.06	2izm	1523.30	1l9a	2399.53
3iev	2535.77	3l25	1917.63	3vjr	1532.32	4yco	2418.71
3aev	2621.48	1r9f	1941.33	1jbr	1535.34	4yvj	2482.18
3bx2	2664.32	1ytu	1984.41	2l3c	1572.58	1yvp	2486.13
1m8y	2736.97	1feu	1995.10	2lbs	1574.75	4gcw	2551.63
4ed5	2737.86	1rc7	2147.24	1jid	1588.81	2csx	2559.79
4b3g	2739.63	5c9h	2224.48	1f6u	1641.41	1lng	2564.27
3bx3	2855.19	1xok	2254.63	2mfh	1681.74	2azx	2595.73
4f02	2857.18	2az0	2479.31	2n3o	1700.75	3kfu	2602.05
3bsx	2913.45	2ykg	2498.78	2xli	1709.84	1ser	2628.90
2mjh	3010.59	1di2	2532.63	4qi2	1731.35	1dk1	2695.08
1g2e	3013.33	2zi0	2590.05	1t4l	1747.47	1mms	2708.31
4bs2	3019.96	2zko	2716.03	1u1y	1810.38	1h4q	2758.39
1b7f	3140.10	4s3n	2750.90	2izn	1830.51	4x4n	2778.11
1cvj	3174.74	1q2r	3004.51	5f5h	1843.62	1efw	2799.95
3k49	3255.33	3eqt	3004.98	2qux	1849.24	3am1	2831.28
4jng	3272.48	5ed1	3091.22	5fj4	1897.66	5aox	2837.74
1fnx	3287.96	5ed2	3159.26	2mfg	1913.00	2r8s	2839.40
5l2l	3323.78	4ig8	3211.47	4c4w	1929.58	1h3e	2921.01
5i9d	3328.99	4rwn	3266.16	3agv	1929.72	4o26	2992.64
5wty	3422.94	2gjw	3312.85	1d6k	1936.69	4yye	3022.84
3qjj	3439.01	4wtd	3332.42	1zbh	1937.68	2ihx	3147.68
4j1g	3451.96	4oog	3339.40	1s03	1941.71	5hc9	3152.48
5i9h	3571.54	1wne	3351.17	1urn	1964.44	1mzp	3207.20
4kxt	3607.76	5f8g	3691.64	1g70	1988.70	4qei	3327.36
4ill	3729.51	5jc3	3845.86	1etf	2007.67	2rfk	3432.37
5aor	3768.67	3bsn	4086.16	4c8z	2009.43	3adb	3685.21
4kre	3781.11	4gha	4137.19	1i6u	2011.25	5ccb	3686.73
2mgz	3808.43	4k4u	4318.41	2l2k	2026.25	2fmt	3687.03
5i4a	3843.87	4k4x	4357.67	2jpp	2030.00	5axm	3715.76
5t7b	3978.44	5jc7	4404.94	4pdb	2034.03	5b63	3903.89
2km8	4007.34	4k50	4581.49	2mff	2044.95	4x4u	3905.14
4krf	4122.68	3ol6	4651.37	4zld	2075.86	2bte	3909.16
4f3t	4251.63	4w5r	4771.04	5m0i	2079.97	1r3e	3930.32
4m59	4521.51	5jaj	5180.74	1ec6	2080.51	5hr6	3964.70

4pmw	4565.60	2nuf	6080.68	4tuw	2098.38	2zm5	4267.63
2ix1	4619.17	4z4g	6293.63	1a1t	2146.15	3nmu	4340.45
4h5p	4845.13	4z4e	6305.96	3oij	2162.17	2xd0	4763.68
4qqb	5209.18	4z4c	6413.50	1aud	2194.88	5ah5	4822.51
3qjl	7603.42	4z4d	6646.96	2mfc	2195.61	4rmo	4845.10
4csf	8882.96	4w5o	6982.82	3oin	2200.63	4rdx	4875.05
2gic	11510.67	4m30	7018.04	4l8h	2205.56	1g59	4899.79
3ptx	12649.30			1ooa	2207.72	1asy	4914.66
4u7u	17676.80			2fy1	2220.20	1c0a	4941.19
1c9s	22384.87			1rkj	2220.37	2zzm	4979.98
				1biv	2222.81	1u0b	5040.10
				2b3j	2226.50	3amt	5228.98
				2mfe	2238.67	3eph	5242.13
				4c7o	2312.96	2bh2	5347.16
				1zbn	2318.93	5id6	5473.59
				1exy	2415.41	1g1x	5512.14
				1l1c	2431.46	1ffy	5512.26
				3bt7	2432.81	2zue	5637.63
				4c8y	2444.24	1qf6	5676.90
				4ato	2459.02	4wsb	5828.47
				1ull	2462.68	2v3c	5857.30
				1zho	2495.40	1gax	5860.81
				1fje	2521.85	5m3h	5885.37
				2vpl	2544.75	3akz	5938.13
				2hgh	2573.03	4n0t	5999.08
				1vfg	2580.76	1euy	6415.90
				2dr7	2684.51	1f7u	6711.74
				1a9n	2725.71	4wrt	6971.49
				3siu	2758.67	3zgz	7113.05
				484d	2777.59	3hjw	7119.48
				2ozb	2798.02	5amq	7211.30
				4al5	2814.41	5wtk	10047.84
				3sn2	2948.02	4by9	10438.62
				2i82	2961.07	4zt0	12053.09
				3rw6	3053.83		
				1ze2	3118.31		
				1k8w	3124.82		
				4kr6	3145.26		
				3snp	3159.52		
				3ouy	3455.68		
				2y8w	3516.12		
				3ovb	3546.95		
				3dh3	3579.57		
				5f9f	3834.56		
				4lgt	3854.88		
				3ts2	3959.95		
				3trz	4207.38		
				3ts0	4415.84		
				4l8r	4698.59		
				2hvy	4753.72		
				3a6p	4919.52		
				3iab	5611.95		
				5jbg	6031.26		
				4pkd	6270.17		
				1mji	6309.25		

Table S2. Experimental alanine scanning data set extracted from dbAMEPNI (filter: nucleic acid = RNA; minimal 5 data pairs per PDB ID) and calculated *PRI HotScore interaction scores* (*IS*).

PDB ID	Variation	*ΔΔG* (kcal·mol^{-1})	*IS*
1asy	N117A	0.88	0.83
	R119A	1.29	3.18
	Q121A	1.30	3.11
	F127A	2.09	3.56
	Q138A	1.42	1.71
	K142A	0.86	1.85
	K155A	0.76	1.59
	K180A	1.52	0.68
	S181A	1.05	0.41
	E188A	1.56	0.14
	D210A	1.41	0.21
	N227A	0.81	1.44
	S301A	0.30	0.02
	N328A	0.61	1.43
	S329A	0.81	0.60
	T331A	1.11	0.60
	H334A	1.29	3.23
	S423A	-0.11	0.10
	T424A	0.81	0.41
	K553A	1.31	2.13
1aud	K49A	1.45	1.28
	M50A	1.10	3.53
	Q53A	4.85	1.68
	F55A	5.50	2.34
	S90A	2.20	0.33
1jbs	D40A	0.21	0.72
	K42A	0.27	0.90
	H49A	0.84	1.37
	T52A	0.85	0.90
	R65A	0.51	1.16
	K110A	1.44	1.78
	K111A	1.81	1.28
	K113A	0.73	1.22
	Q141A	0.42	1.17
	D143A	-0.52	0.64
2ix1	Y253A	0.88	3.34
	Y313A	0.51	1.47
	F358A	0.22	4.05
	R500A	0.27	3.76
	E542A	-1.35	0.12
4ed5	N21A	0.64	0.84
	T90A	0.67	0.04
	R97A	1.49	1.33
	R136A	0.96	2.02
	R147A	0.87	1.01
5gxh	W14A	2.17	1.51
	Y15A	1.19	3.30
	E197A	1.66	0.53
	F381A	2.16	1.93
	Y474A	0.02	2.27
5h1k	N13A	1.04	0.95
	W14A	3.05	1.12
	Y15A	2.02	1.18
	R33A	1.54	1.10
	M357A	2.08	1.28
	R359A	3.12	1.20
